# Serum sirtuin1: a potential blood biomarker for early diagnosis of Alzheimer’s disease

**DOI:** 10.18632/aging.205015

**Published:** 2023-09-22

**Authors:** Jia Wang, Fang Zhou, Chang-E Xiong, Gui-Ping Wang, Lin-Wanyue Chen, Yu-Tong Zhang, Shi-Ge Qi, Zhi-Hui Wang, Can Mei, Yu-Jia Xu, Jian-Bo Zhan, Jing Cheng

**Affiliations:** 1 School of Public Health, Wuhan University of Science and Technology, Wuhan 430065, Hubei, China; 2 Hubei Provincial Center for Disease Control and Prevention, Wuhan 430079, Hubei, China; 3 Hubei Province Key Laboratory of Occupational Hazard Identification and Control, Wuhan University of Science and Technology, Wuhan 430065, Hubei, China; 4 Chinese Center for Disease Control and Prevention, Beijing 102206, China

**Keywords:** serum, biomarker, early diagnosis, Alzheimer’s disease

## Abstract

Background: Sirtuin 1, a nicotinamide adenine dinucleotide-dependent deacetylase that is highly expressed in the hippocampus and anterior cortex tissues related to Alzheimer’s Disease pathology, can cross the blood-brain barrier and is a promising biomarker.

Methods: A 1:1:1 case-control study was conducted and serum fasting blood glucose, triglyceride, total cholesterol, high-density lipoprotein cholesterol, low-density lipoprotein cholesterol, SIRT1, IL-6, Aβ1-42, T-tau and P-tau-181 levels were evaluated in blood samples of 26 patients form the Alzheimer’s Disease group, 26 patients form the mild cognitive impairment group, and 26 individuals form the normal control group. Receiver operator characteristic curves were used to evaluate the diagnostic significance.

Results: Serum SIRT1 level was significantly down-regulated in the mild cognitive impairment patients and Alzheimer’s Disease patients compared with that in the normal control group (*P*<0.05). ROC curve analysis demonstrated that SIRT1 was a promising biomarker to distinguish Alzheimer’s Disease patients from the mild cognitive impairment patients and the normal control group. In addition, SIRT1 was estimated to perform well in the diagnosis of Alzheimer’s Disease ([AUC] = 0.742).

Conclusions: In summary, the present study suggested that serum SIRT1 might be an early promising diagnostic biomarker for Alzheimer’s Disease.

## INTRODUCTION

According to the latest statistics of the World Health Organization (WHO), Alzheimer’s Disease (AD) is the seventh cause of death among all diseases in the world [1]. AD is the most common senile neurodegenerative disease characterized by cognitive dysfunction and behavioral changes, and is the main cause of senile dementia [2, 3]. Epidemiological data forecast that there are approximately 50 million people living with dementia worldwide, and nearly 60% of them live in low- and middle-income countries up to now. There are 10 million new cases a year. The proportion of people aged 60 years and over with dementia is estimated to be 5–8%, and the total number of people with dementia will reach 82 million by 2030 and 152 million by 2050 [4, 5].

Mild cognitive impairment (MCI) is an early stage of preclinical AD [6]. According to the statistics, there is 10–20% of MCI patients progressing to AD every year. AD is irreversible, yet MCI can be reversed [7]. Therefore, early identification and diagnosis of MCI is of great significance. At present, the diagnosis and treatment of AD or MCI are still very insufficient worldwide, especially in China, where the consultation rate of AD patients is relatively low.

The most accurate way to diagnose Alzheimer’s disease is to perform brain dissection on the patient’s brain tissue and determine whether the subject has typical pathological manifestations of Alzheimer’s disease, such as neuroinflammatory plaques and neurofibrillary tangles, to determine whether the subject has Alzheimer’s disease [8, 9]. However, due to the non-feasibility of such methods, to diagnose and conclude the conditions, medical practitioners mainly depend on clinical symptoms, physical examination, MMSE, positron emission tomography (PET) of tracer molecules [10] and analysis of cerebrospinal fluid (CSF) proteins [11] and use tests to examine a patient’s mental ability [9, 12, 13]. However, the sensitivity and specificity of such tests are usually not high enough; thus, these tests cannot be used to make an effective and accurate judgment of the disease. On the other hand, the procedures are complicated and often affected by many factors. The newer genetic tests [14] and CSF biochemical tests in recent years cannot be popularized in hospitals at all levels, due to their inconvenient procedures and expensive examination cost, thus limiting their clinical application. MoCA is a promising tool, but its specificity for detecting early AD is rather low [15]. As a result, less traumatic accessible and inexpensive blood tests are widely accepted and have become more and more charming in AD diagnosis. Blood samples are easy to obtain, stable, less traumatic, and can avoid the decline in physical function caused by pathological punctures; these advantages determine the great superiority of blood biomarkers [16–20]. Therefore, the exploration of biomarkers for early identification and diagnosis of AD in peripheral blood has become a research hotspot. At present, the plasma biomarkers that have been proved to be strongly correlated with AD include plasma T-tau, P-tau-181 [21], p-tau217, p-tau231 [22], NFL [23] and Peripheral inflammatory biomarkers [24]. Because of the consistency of these proteins, the plasma T-tau, P-tau-181, Aβ42, and NFL proteins are used in clinical trials and studies. In recent years, with the improvement of the sensitivity and specificity of enzyme-linked immunosorbent assay (ELISA), it has been gradually used to detect Aβ1-42, T-tau, and P-tau-181 in peripheral blood [25], which is in conformity with our previous findings [22].

SIRT1 is a NAD+-dependent deacetylase with neuro-protective functions [26]; it is highly expressed in the hippocampus and anterior cortex tissues related to AD pathology and can access all body tissues after crossing the blood-brain barrier [27]. Recently, a host of studies have reported the protective role of SIRT1 in aging-related diseases, which can participate in the regulation of cell differentiation, apoptosis, oxidative stress, autophagy and other processes by playing a deacetylation role [28, 29]. Liu, L., et al. [30] found that the SIRT1 level was down-regulated in the brain of AD patients during progression from the early stage to the late stage. Therefore, serum SIRT1 protein is regarded as a potential biomarker for the early diagnosis of AD. In the present study, based on our previous research work [31], we aimed to detect serum SIRT1, Aβ1-42, T-tau, and P-tau-181 protein levels in AD, MCI patients, and normal controls (NCs), and assess their potential as early diagnostic biomarkers for AD.

## RESULTS

### Detection of blood-related indexes

### Detection of blood biochemical indexes

As shown in Table 1.

**Table 1:** Comparison of blood biochemical parameters in the case and control groups (mean±SD).

**Variables (mmol/L, mean±SD)**	**NC (n=26)**	**MCI (n=26)**	**AD (n=26)**	P*	P**	P***
FBG	4.53±0.98	8.09±3.99	4.75±1.99	0.612	0.000	0.000
TC	5.26±1.26	4.81±1.48	5.46±1.18	0.582	0.264	0.086
TG	1.44±0.55	1.81±1.08	1.14±0.54	0.098	0.105	0.007
LDL-C	2.61±0.87	2.85±1.69	3.68±2.02	0.023	0.508	0.110
HDL-C	1.68±0.37	1.68±0.47	1.53±0.39	0.178	0.982	0.182

Note: P* represents the comparison between AD group and NC group, P** represents the comparison between MCI group and NC group and P***represents the comparison between MCI group and AD group. FBG, fasting blood glucose; TC, total cholesterol; TG, triglyceride; LDL-C, Low-Density Lipoprotein Cholesterol; HDL-C, High-Density Lipoprotein Cholesterol.

### Serum assays for SIRT1, IL-6, Aβ1-42, T-Tau, and P-tau-181 protein

**Figure 1:**
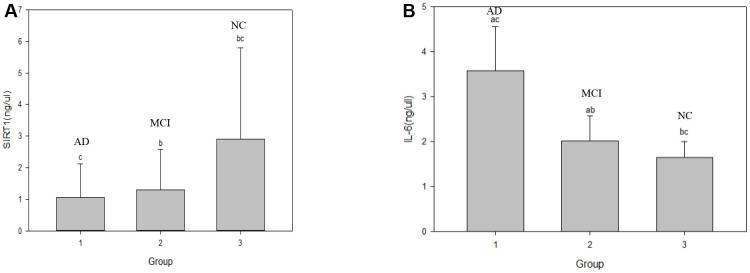
**Comparison of serum SIRTI and IL-6 levels in the NC, MCI and AD groups.** Note: a represents *P, b represents **P and c represents ***P, *P represents the comparison between AD and MCI groups; **P represents the comparison between MCI and NC groups; ***P represents the comparison between the AD and NC groups. (**A**) graph shows the changes in serum SIRT1 level in the three groups respectively. (**B**) graph shows the changes in serum IL-6 level in the three groups respectively. NC: normal control, MCI: mild cognitive impairment, AD: Alzheimer’s Disease.

**Figure 2:**
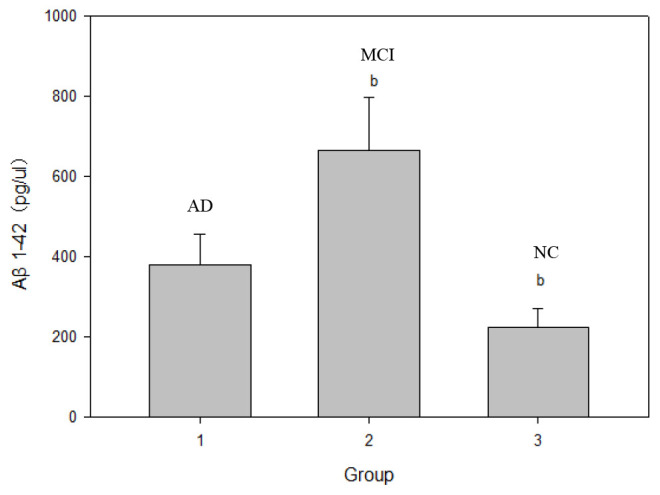
**Comparison of serum Aβ1-42 protein levels in NC, MCI and AD groups.** Note: b represents **P; **P represents the comparison between MCI and NC groups.

**Figure 3:**
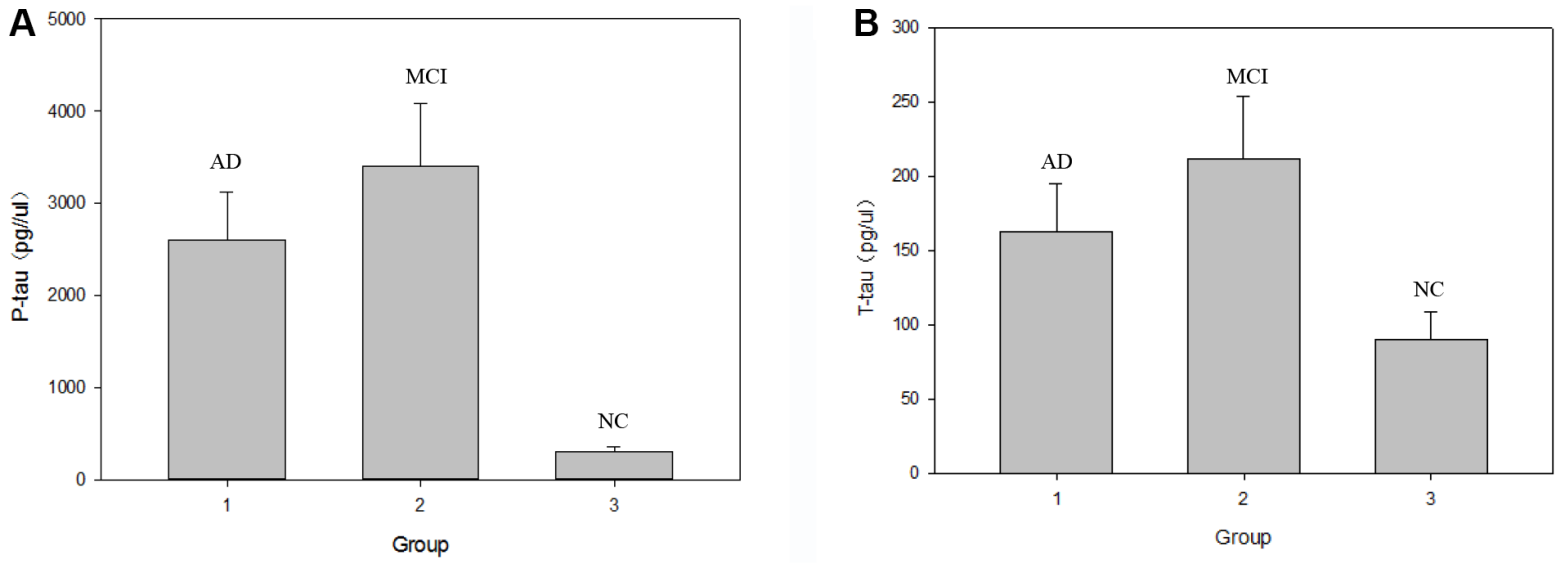
**Comparison of serum P-tau-181 and T-tau protein levels in the NC, MCI and AD groups.** Note: (**A**) graph shows the changes in serum P-tau-181 level in the three groups respectively. (**B**) graph shows the changes in serum T-tau level in the three groups respectively.

As shown in Table 2, serum SIRT1 protein levels were successively down-regulated in the NC, MCI, and AD groups. Compared with that in the NC group, the serum SIRT1 level in the MCI and AD groups was significantly down-regulated, and the difference was statistically significant (*P*< 0.05). As shown in Figure 1, the level of inflammatory factor IL-6 was inversely correlated with the change in SIRT1 levels. Compared with that in the NC group (1.65±0.35 ng/μL), the serum IL-6 levels in the MCI group (2.02±0.56 ng/μL) and the AD group (3.58±0.98 ng/μL) were significantly up-regulated (*P*< 0.05), as shown in Figure 2, and the serum Aβ1-42 level in the MCI group was significantly up-regulated (*P*< 0.05); that in the AD group was also up-regulated, but the difference was not significant (*P*> 0.05). In the NC, MCI, and AD groups, the serum Aβ1-42 level showed a trend of up-regulation first and then down-regulation. As shown in Figure 3, compared with those in the NC group, there was no significant difference in the serum P-tau-181 and T-tau levels among the NC, MCI, and AD groups (*P*> 0.05).

**Table 2:** Comparison of serum protein levels in the case and control groups (mean±SD).

**Variables (mean±SD)**	**AD(n=26)**	**MCI(n=26)**	**NC(n=26)**	P*	P**	P***
SIRT1(ng/μl)	1.06±0.471	1.29±0.21	2.90±2.01	0.441	0.000	0.000
IL-6(ng/μl)	3.58±0.98	2.02±0.56	1.65±0.35	0.000	0.011	0.000
Aβ1-42(pg/μl)	380.18±76.04	665.87±133.17	225.43±45.09	0.187	0.018	0.200
P-tau-181(pg/μl)	2598.4±519.68	3404.7±680.94	298.20±59.64	0.104	0.394	0.454
T-tau(pg/μl)	162.37±32.47	211.38±42.28	90.43±18.09	0.505	0.067	0.237

Note: *P represents the comparison between AD and MCI groups; **P represents the comparison between MCI and NC groups; ***P represents the comparison between the AD and NC groups.

### Diagnostic performances of SIRT1, IL-6, Aβ1-42, P-tau-181, and T-tau protein for AD

**Figure 4:**
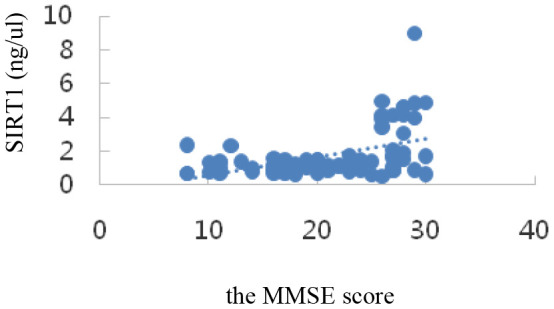
Correlation analysis of SIRT1 and MMSE.

**Figure 5:**
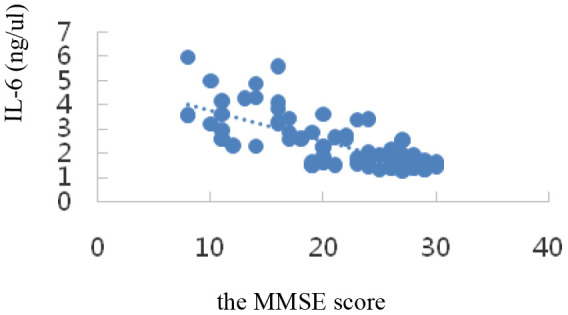
Correlation analysis of IL-6 and MMSE.

**Figure 6:**
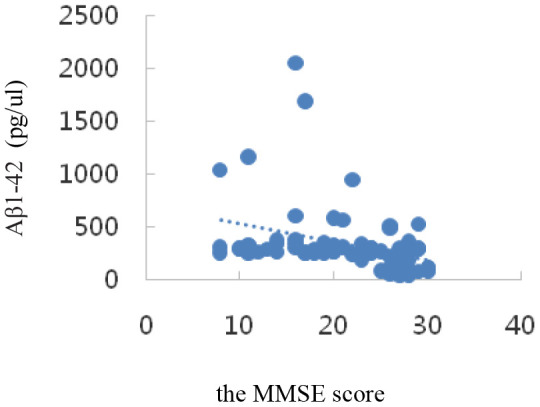
Correlation analysis of Aβ1-42 and MMSE.

**Figure 7:**
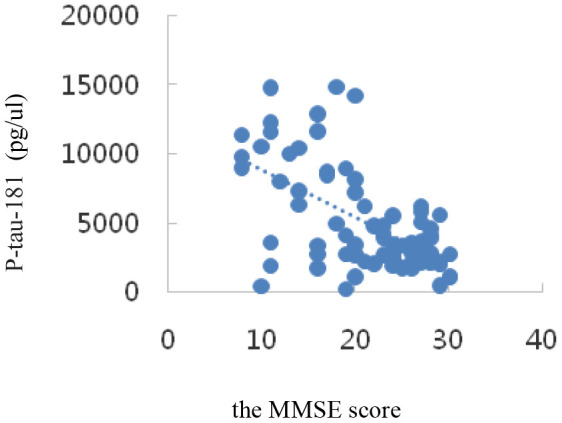
Correlation analysis of P-tau-181 and MMSE.

**Figure 8:**
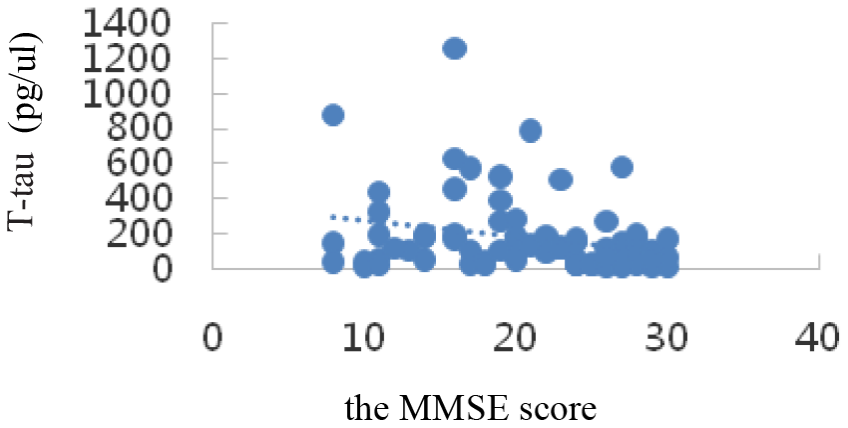
Correlation analysis of T-tau and MMSE.

Correlation analysis showed that serum SIRT1 levels were positively correlated with MMSE scores (r_s_=0.47, *P*=0.000); IL-6, Aβ1-42, T-Tau, and P-tau-181 levels were inversely correlated with MMSE scores (r_I_=-0.73, P=0.000; r_A_=-0.34, *P*=0.002; r_T_=-0.58, *P*=0.000; r_P_=-0.27, *P*=0.015); all differences were statistically significant (*P*<0.05). The results are shown in Table 3 and the scatter plots are shown in Figures 4–8.

**Table 3:** Correlation of serum SIRT1, IL-6, Aβ1-42, T-Tau and P-tau-181 levels with MMSE scores.

**Variables**	**r**	***P*(n=78)**
SIRT1(ng/μl)	0.47	0.000
IL-6(ng/μl)	-0.73	0.000
Aβ1-42(pg/μl)	-0.34	0.002
P-tau-181(pg/μl)	-0.58	0.000
T-tau(pg/μl)	-0.27	0.015

**Figure 9:**
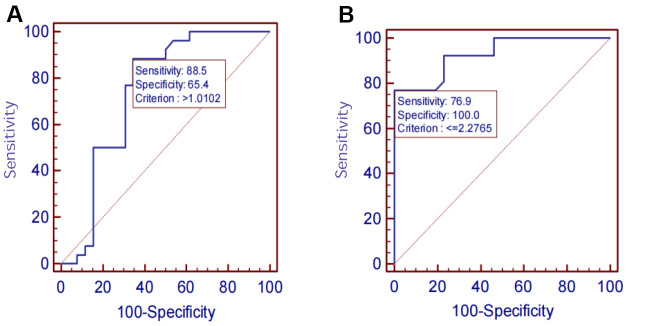
(**A**) SIRT1 ROC curve. (**B**) IL-6 ROC curve.

**Figure 10:**
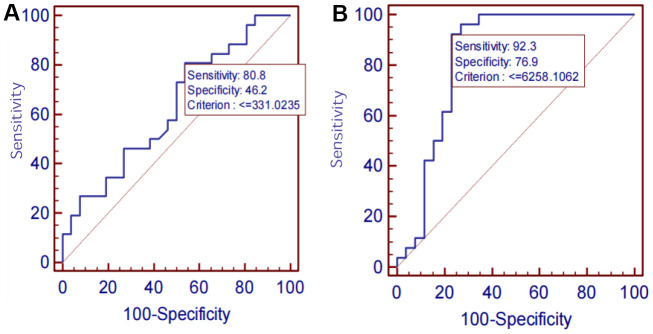
(**A**) Aβ1-42 ROC curve. (**B**) P-tau-181 ROC curve.

**Figure 11:**
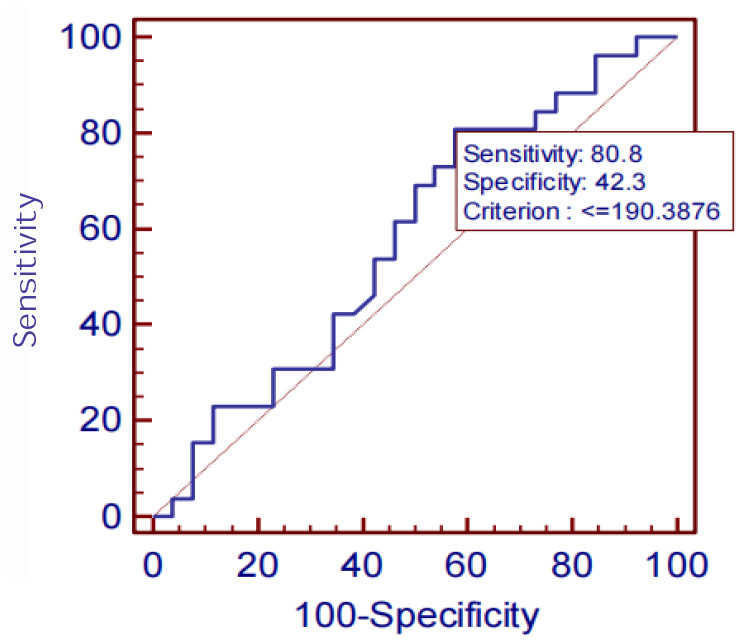
T-tau ROC curve.

The diagnostic performances of serum SIRT1, IL-6, Aβ1-42, P-tau-181, and T-tau for AD were evaluated by ROC curve analysis. The AUC, sensitivity, specificity, and all cut-off values of SIRT1, IL-6, Aβ1-42, P-tau-181, and T-tau levels were determined using ROC analysis and were summarized in Table 4. SIRT1 predicted the presence of AD with an AUC of 0.742 (95% CI: 0.60–0.85), 88.5% sensitivity, and 65.4% specificity. The ROC curves are shown in Figures 9–11.

**Table 4:** ROC curve analysis of serum protein indicators (n=78).

**ROC indicator**	**Criterion**	**Se(%)**	**Sp(%)**	**AUC**	**95%CI**	***P***
SIRT1(ng/μl)	> 1.0102	88.5	65.4	0.742	0.60-0.85	0.000
IL-6(ng/μl)	≤2.2765	76.9	100	0.930	0.82-0.98	0.000
Aβ1-42(pg/μl)	≤331.0235	80.8	46.2	0.629	0.48-0.76	0.098
P-tau-181(pg/μl)	≤6258.1062	92.3	76.9	0.831	0.70-0.92	0.000
T-tau(pg/μl)	≤190.3876	80.8	42.3	0.578	0.43-0.71	0.338

Note: ROC, receiver operator characteristic.

## DISCUSSION

### The key findings of the study

The silent information regulator sirtuin 1 (SIRT1) protein is a histone deacetylase, which links the metabolic state of cells with the regulation of gene expression and many processes related to cancer (such as apoptosis, oxidative stress, neuronal autophagy, immune response and so on) [32, 33]. Sirtuin 1 is highly expressed in the hippocampus and anterior cortex, which are associated with Alzheimer’s disease (AD) pathology. Overexpression or elevation of SIRT1 concentration can reduce Aβ aggregation and degrade tau. Therefore, lack of sirtuin expression in the hippocampal neurons will impair cognitive function, including recent memory and spatial learning [34]. Previous studies have shown that the reduction of SIRT1 level can lead to Alzheimer’s disease by promoting Aβ aggregation and tau hyperphosphorylation [35, 36]. Pradhan, R. et al. reported that the sirtuins family was composed of seven members. Compared with MCI and GC, the sirtuins in AD group were significantly reduced, and the levels of SIRT1, SIRT3 and SIRT6 (mean±SD) were significantly reduced. ROC analysis showed that for AD and MCI had high sensitivity and specificity [37]. In addition, Jiao, F. and Z. Gongamong also showed that seven sirtuins, SIRT1, play an significant regulatory role in a wide range of physiological processes, including oxidative stress, regulation of synaptic plasticity, metabolism as well as cancer [38]. Cui, Z., et al., and Buglio, D.S., et al. reported that SIRT1 may provide a new therapeutic direction for the treatment of age-related diseases by inhibiting oxidative stress, reducing inflammatory response and restoring mitochondrial dysfunction [39]. There are some studies demonstrating that SIRT1 regulates the expression of proinflammatory cytokines, for instance, tumor necrosis factor (TNF), interleukin-1 β (IL-1β), and interferon γ (IFN-γ) during microglial activation [40], as well as alleviates degeneration of dopaminergic neurons [41]. Yin, Z., et al. and Yin, X., et al. showed that SIRT1 reduces the expression of Aβ [42] and tau [43], which are associated with AD. Mitochondrial dysfunction is involved in aging and various degenerative diseases, and SIRT1 can regulate mitochondrial biosynthesis and metabolism, repair mitochondrial loss and maintain mitochondrial stability [44]. Furthermore, Shi, L. et al. revealed that SIRT1 directly controls the activity of proliferator-activated receptor γ coactivator 1α (PGC-1α) through phosphorylation and deacetylation to regulate mitochondrial function [45]. These findings illustrate that SIRT1 is a potential therapeutic target for AD and remains to be further revealed.

Therefore, our study screened sporadic AD and MCI patients of community origin among the elderly aged 60 years and above in Hubei Province through a cross-sectional study, and then a 1:1:1 case-control study was conducted in 26 AD patients, 26 MCI patients, and 26 NC subjects, according to gender and age (±2 years). A total of 26 NC subjects were selected and their peripheral venous blood was collected to find a more stable biomarker for the early diagnosis of AD by detecting the levels of serum SIRT1, IL-6, T-tau, P-tau-181, and Aβ1-42 proteins in the three groups of subjects, to provide ponderable help for the early diagnosis of AD.

In the transition from MCI to AD, namely, the AD stage, the opposite changes in blood glucose and lipid levels may occur, which is consistent with Shang Ying’s report [46], that is, the relationship between blood glucose level and hyperlipidemia and Alzheimer’s disease may not be positively correlated. Compared with that in the NC group, the serum Aβ1-42 level in the MCI group was significantly up-regulated (*P*< 0.05); that in the AD group was also up-regulated, but not statistically significantly (*P*> 0.05), which indicated a trend of up-regulation first and then down-regulation among NC, MCI, and AD groups. Compared with those in the NC group, the serum P-tau-181 and T-tau levels showed a trend of up-regulation first and then down-regulation in the MCI and AD groups, but not statistically significantly (*P*> 0.05). Possible reasons are as follows: on the one hand, when normal people first manifest cognitive impairment in the process of transforming to MCI, the levels of Aβ1-42, T-tau, and P-tau-181 proteins in the brain increase sharply. The concentration of these proteins in the blood is greatly diluted owing to the existence of the blood-brain barrier [47]. Once there is progression to AD, the levels of the three proteins stabilize at a low level. On the other hand, the concentration of Aβ in peripheral blood is affected by the small amount of Aβ produced by platelets, so its concentration change in CSF is not synchronized [48]. It has been reported [49] that plasma Aβ1-42 protein concentration was down-regulated significantly in both AD and MCI patients. However, studies by some scholars have shown [50] that the changes in plasma Aβ1-42 protein concentration in AD and MCI patients show staged differences, that is, the concentration of Aβ1-42 down-regulates with progression from the MCI stage to the AD stage, but there is no significant difference with progression from the normal state to the MCI stage. It has also been reported [51] that the concentration of plasma Aβ1-42 protein in AD patients is significantly higher than that in the NC group. In short, the research results of Aβ1-42 in the peripheral blood of patients with AD and early MCI by different researchers are inconsistent, or even opposite, and Aβ40 in plasma is also inconsistent [52]. However, serum Aβ1-42, T-tau, and P-tau-181 protein levels were inversely correlated with MMSE scores, which was consistent with previous findings. The levels of these special proteins were up-regulated or down-regulated sharply in the MCI stage, but were down-regulated or up-regulated sharply in the AD stage. This disorder in the level of specific protein changes suggests the importance of studying biomarkers of MCI in the early stages of AD, the intermediate stage of the disease.

Our data demonstrated that the levels of SIRT1 decreased significantly in AD patients. According to the detection of serum SIRT1, IL-6, T-tau, P-tau-181, and Aβ1-42 proteins, the results showed that in the NC, MCI, and AD groups, the serum SIRT1 protein levels were down-regulated successively, and they were (2.90±2.01), (1.29±0.21), and (1.06±0.471) ng/μL, respectively. Compared with that in the NC group, the serum SIRT1 levels in the MCI and AD groups were significantly down-regulated, and the difference was statistically significant (*P*< 0.05). Interestingly, the serum SIRT1 protein levels in the case groups and the control group were positively correlated with the MMSE score (r_S_ =0.47, *P*=0.000), and the difference was statistically significant (*P*< 0.05), which was consistent with a previous report [53]. The levels of IL-6 were up-regulated with the progression of disease from NC to MCI and then to AD: they were (1.65±0.35), (2.02±0.56), and (3.58±0.98) ng/μL, respectively. Compared with that in the NC group, the serum IL-6 levels in the MCI and AD groups were significantly up-regulated (*P*< 0.05). These results suggest that inflammation is an important process of MCI to AD transformation. It was also found that serum IL-6 level was inversely correlated with SIRT1 level (r_I_=-0.73, *P*=0.000), and the difference was statistically significant (*P*< 0.05). Studies have shown that [54] compared with cognitively healthy controls, AD patients may have inflammatory signs in the hypothalamus and hippocampus (as shown by T2 hyperintensity). T2 hyperintensity in the hypothalamus and hippocampus is positively correlated with plasma IL-6 concentration and inversely correlated with MMSE score, which is very important for the production of AD. Chen X. [54] et al. showed that the genetic variation and deacetylation function of SIRT1 are directly related to the occurrence of inflammation, and play a role in inhibiting inflammation through the NF-κB pathway, thereby inhibiting the expression of IL-6.

On analyzing the sensitivities, specificities, and AUCs of SIRT1, IL-6, Aβ1-42, P-tau-181, and T-tau in serum, the sensitivities and specificities of the serum proteins decreased successively: P-tau-181 (92.3%, 76.9%), SIRT1 (88.5%, 65.4%), Aβ1-42 (80.8%, 46.2%), T-tau (80.8%, 42.3%), IL-6 (76.9%, 100.0%). However, the AUC was IL-6 >P-tau-181> SIRT1 > Aβ1-42 > T-tau. ROC analysis showed the AUC, sensitivity and specificity of screening AD using P-tau-181 were all higher than that using SIRT1. However, the serum P-tau-181 levels was no significant difference among the NC, MCI, and AD groups (*P*> 0.05). The ROC curve is only an estimate, and serological results are more reliable, so we did not consider the diagnostic performance of P-tau-181. The levels of SIRT1, IL-6, and P-tau-181 and MMSE were considered statistically significant (*P*< 0.05), but the levels of Aβ1-42 and T-tau were not considered statistically significant (*P*> 0.05). The results showed that the sensitivity and specificity of screening AD were best when the critical value of the MMSE was above 18. However, there is a certain subjective bias in the scale, which is not as accurate as the blood index. The performances of SIRT1 combined with other markers of AD require further study in the future.

### Strength and limitations

Our study is the first cross-sectional and case-control study of sporadic AD and MCI in Hubei province. In addition, our results provide more meaningful biomarkers for the early diagnosis of AD, and also provide new ideas for the basis of early diagnosis. However, to some extent, our study also had some limitations, and there is a need to conduct validation with a larger sample size of AD patients from different sources in the future to obtain more relevant and reliable evidence.

## CONCLUSIONS

In a word, the present study revealed that serum SIRT1 presumably was an early promising diagnostic biomarker for AD.

## MATERIALS AND METHODS

### Source of subjects

A cross-sectional and case-control study were performed. The case groups and the control group were all from Qichun County, Huanggang City, and Dongbao District, Jingmen City, Hubei Province. The case groups were AD and MCI patients aged ≥ 60 years, with 26 cases in each group. Their mean ages were (70.02 ± 4.68) and (69.83 ± 4.84) years, respectively, while the control group consisted of 26 healthy elderly people aged ≥ 60 years, with a mean age of (70.17±5.99) years. As shown in Table 5A. General information of case group and control group, including education level, alcohol drinking, tea drinking, physical exercise, and body mass index (BMI), marital status, disease history, and smoking were collected and summarized in Table 5B. The research protocol of the experiment was approved by the Ethics Committee of the Medical College of Wuhan University of Science and Technology, and informed consent was obtained from all individual participants of the present study.

**Table 5A:** Age, gender and MMSE score of case group and control group.

**Variables**	**AD (n=26)**	**MCI (n=26)**	**NC (n=26)**	***P***
Age (Years, mean±SD)	70.17±5.99	69.83±4.84	70.02±4.68	0. 823
Gender (M/F)	11/15	11/15	11/15	1.00
MMSE (mean±SD)	28.00±1.17	21.83±1.97	13.65±3.34	0.001

Note: Mini-mental State Examination (MMSE); Equilibrium analysis showed that there were no statistically significant differences in age and gender between the AD and MCI case groups and the respective control group (*p*> 0.05), indicating that the groups were comparable. AD, Alzheimer’s Disease; MCI, mild cognitive impairment, NC, normal control.

**Table 5B:** General information of case group and control group.

**Variables**	**NC (n=26)**	**MCI (n=26)**	**AD (n=26)**	***P***
Education n (%)				0.001
University	0(0)	4(15.38)	0(0)	
High school	5 (19.23)	2(7.69)	0(0)	
Junior middle school	10(38.46)	5(19.23)	0(0)	
Primary school	7(26.92)	10(38.46)	1(3.85)	
Illiterate	4(15.38)	5(19.23)	25(96.15)	
Marital status n (%)				0.211
Married	21(80.77)	21(80.77)	16(61.54)	
Divorce	0(0)	1(3.85)	0(0)	
Widowed	5(19.23)	4(15.38)	8(30.77)	
Unmarried	0(0)	0(0)	2(7.69)	
Disease history n (%)				0.768
Diabetes	2(7.69)	1(3.85)	1(3.85)	0.582
Hypertension	7(26.92)	10(38.46)	14(53.85)	0.062
Stroke	1(3.85)	4(15.38)	0(0)	0.652
Smoking n (%)				
Never smoked	20(76.92)	20(76.92)	19(73.08)	
Used to smoke	1(3.85)	3 (11.54)	3 (11.54)	
Smoking now	5(19.23)	3 (11.54)	4(15.38)	
Drinking n (%)				0.031
Never drink	20(76.92)	19 (73.08)	24(92.31)	
Go to drink	0(0)	4 (15.38)	0(0)	
Drink now	6(23.08)	3 (11.54)	2(7.69)	
Drinking tea n (%)				0.001
Never drink tea	11(42.30)	10(38.46)	24(92.31)	
Drink tea in the past	5(19.23)	4(15.38)	0(0)	
Now drink tea	10(38.46)	12(46.15)	2(7.69)	
Physical exercise n (%)				0.001
Low intensity	14(53.85)	4(15.38)	1(3.85)	
Medium intensity	10(38.46)	2(7.69)	0(0)	
High intensity	2(7.69)	20(76.92)	25(96.15)	
BMI (mean±SD)	24.15±3.15	23.35±2.52	20.96±2.92	0.001

Note: (1) * *P* ≤ 0.05 indicates statistical significance.

(2) Low-intensity exercise: regular exercise for less than 1 hour, such as office workers; medium-intensity exercise: exercise for more than 1 hour and less than 2 hours, such as drivers; high-intensity exercise: exercise for more than 2 hours per week, such as farmers and steelmakers workers, etc.

(3) BMI = weight (kg)/height (m)^2^.

### Inclusion and exclusion criteria

### Inclusion and exclusion criteria for the case group

The results are shown in Table 6A.

**Table 6A:** Inclusion and exclusion criteria for the case group.

**Inclusion criteria for the AD group**	**Inclusion criteria for the MCI group**	**Exclusion criteria for the case group**
(1) Aged 60 years and above; (2) MMSE assessment: illiteracy ≤ 17 points, primary school ≤ 20 points, middle school or above education ≤24 points; (3) Meet the criteria for diagnosis of AD in NIA-AA (2011) [55], including memory impairment; at least one abnormality in aphasia, apraxia (normal motor function), agnosia (normal vision), and executive dysfunction; cognitive dysfunction seriously affecting occupational and social functions; (4) CDR ≥ 1 point; (5) HIS ≤ 4 points; (6) GDS in 21 ~ 30 points; (7) Patients and their family members gave informed consent and voluntarily signed consent forms. (8) Combinating imaging manifestations and diagnosis were positive.	(1) Aged 60 years and above; (2) MMSE: illiteracy > 17, primary school > 20, middle school or above education >24; (3) Meeting the Peterson diagnostic criteria: informant report or patient’s complaint of memory impairment; Normal general cognitive function; Normal daily living ability; Does not meet the diagnostic criteria for dementia; (4) CDR ≤ 0.5 point; (5) HIS ≤4 points; (6) GDS < 21 points; (7) Patients and their family members gave informed consent and signed consent forms voluntarily.	(1) Presence of other types of dementia (such as Vascular dementia (VD) and Parkinson disease (PD)or systemic diseases that can cause dementia; (2) Dementia caused by intracranial space-occupying lesions or other physical and chemical factors, such as brain tumours, brain trauma, inflammatory or demyelinating diseases of the central nervous system, and normal intracranial pressure hydrocephalus; (3) Presence of significant mental illnesses such as depression, schizophrenia, and alcohol abuse or drug use disorders; (4) Patients with severe visual and hearing impairment or other physical diseases and who are unable to cooperate with the completion of the questionnaires and other examinations.

Note: CDR, Clinical Dementia Rating; HIS, Hachinski Ischemic Score; GDS, Geriatric Depression Scale.

### Inclusion criteria and exclusion for the control group

The results are shown in Table 6B.

**Table 6B:** Inclusion criteria and exclusion for the control group.

**Inclusion criteria for the control group**	**Exclusion criteria for the control group**
(1) Age ≥ 60 years; (2) Normal ability of daily living; (3) MMSE ≥ 28, cognitive function normal; (4) No family history of dementia.	(1) MMSE ≤ 28 points; (2) Having other types of dementia, cerebrovascular diseases, such as stroke, meningitis, and other central nervous system diseases.

### Collection, processing, storage and transportation of blood samples

With the informed consent of all the researchers, the serum samples of the case group and the control group were collected. The specific collection methods are as follows:

1. The blood collection code strip was affixed, and the blood collection code was filled in the circulation form of the respondents by special personnel at the registry office, and the blood collection code strip was affixed on the blood collection vessels as required.
2. Collecting 3-4ml venous blood in 5ml disposable vacuum negative pressure collection vessel (red cap tube, with milkWhite glue block), we placed it at room temperature for 30 minutes, centrifuged at 3000r/min for about 15 minutes, and separated the serum in the clean workbench (note: the lid must be tightly covered, affixed with the corresponding label, immediately transferred to -20° C for storage).
3. We collected 3-4ml venous blood in a disposable vacuum negative pressure EDTA-2K anticoagulant collection vessel (purple cap tube). The venous blood was then gently upsided down 6 times, transferred to-20° C within 1 hour for storage, used for plasma isolation.
4. After all blood samples are processed, the numbers must be checked, and a record paper must be attached to each freezing box, indicating the starting number, ending number, lack number, hemolysis number and date of the district (county), township (street) and village (neighborhood committee) where the blood is collected; and attach the signature of the person in charge. In addition, the freezing box number and blood sample type (serum, plasma, whole blood) should be marked on the surface and side of the freezing box. The blood samples were stored at low temperature in the laboratories of each project site and transported to the testing center of Wuhan University of Science and Technology School of Medicine on dry ice within one week.

### Blood biochemical indexes were detected by a microplate reader

Fasting blood glucose (FBG), triglyceride (TG), total cholesterol (TC), high density lipoprotein cholesterol (HDL-C) and low density lipoprotein cholesterol (LDL-C) were measured by a microplate reader in 26 patients with AD, 26 patients with MCI and 26 patients with NCs. The kits were from Zhongsheng Beikong Biotechnology Co., Ltd. (China), and the specific operation methods were strictly in accordance with the kit instructions.

### Serum SIRT1, IL-6, Aβ1-42, T-Tau, and P-tau-181 detection by ELISA

SIRT1, IL-6, Aβ1-42, T-Tau, and P-tau-181 levels in serum samples were measured by ELISA (Elabscience Inc., WH, China), as stated or reported by the manufacturer’s instructions. In simple terms, a microtiter plate coated with capture antibody was incubated with 100-μl serum samples from the case group and NC group for 1 h at 37° C. The detection antibody was added after washing and incubated for 30 min at room temperature. Adequate washing was carried out after each step. Following avidin-horseradish peroxidase-conjugated secondary antibody and TMB substrate solution, stop solution was added to terminate the reaction. Eventually, every protein was determined at a special wavelength using a Model 96 microplate reader. Each sample was analyzed in duplicate.

### Statistical analysis

SPSS 22.0 software (SPSS Inc., Chicago, IL, U.S.A.) was used to analyze all data for statistical significance. All data are presented as mean ±SD. For general demographic data, the χ^2^ test was used for categorical variables and the Mann Whitney–Wilcoxon test was used for continuous variables. ANOVA was performed to ascertain any statistically significant differences between the preoperative and postoperative levels of proteins. Pearson correlation coefficient was used to evaluate the correlation between the two biomarkers. ROC curves drawn assess diagnostic capability. AUC and 95% confidence intervals were used to assess discrimination ability. Values of *P*<0.05 were considered statistically significant.

### Consent for publication

Informed consent for publication of this report and any accompanying images was obtained from the participants

### Availability of data and materials

The datasets used and/or analysed during the current study available from the corresponding author on reasonable request.
